# Membrane-Spanning DNA Nanopores with Cytotoxic Effect**

**DOI:** 10.1002/anie.201405719

**Published:** 2014-10-07

**Authors:** Jonathan R Burns, Noura Al-Juffali, Sam M Janes, Stefan Howorka

**Affiliations:** Department of Chemistry, University College London, Institute of Structural and Molecular Biology, 20 Gordon Street London WC1H OAJ (UK); Lungs for Living Research Centre, Division of Medicine, University College London London WC1E 6JF (UK)

**Keywords:** cancer, cells, DNA origami, nanopores, nucleic acids

Self-assembled DNA nanostructures with chemistry-enabled functionality are of great interest in nanobiotechnology. Herein, chemically modified DNA nanopores are designed to puncture cellular membranes and cause cytotoxicity. The nanopores are assembled from DNA oligonucleotides to form a 2 nm-high hydrophobic belt at one terminus. The belt is composed of charge-neutralized ethyl phosphorothioate (EP) groups which are required to decrease cell viability. The mode by which the pores achieve cell killing is elucidated with confocal microscopy. This study is the first to describe the interaction of DNA nanopores with cells. The work lays the foundation for the future development of cytotoxic agents with cancer type-specificity.

Chemistry can play an important role in expanding the functional repertoire of DNA nanostructures.^1^, ^2^ Designed nanomaterials have been developed by equipping DNA scaffolds^2^, ^3^ with chemical linkages to spatially arrange nanoparticles,^4^ fluorophores,^5^ or proteins.^6^ These DNA nanostructures have been mostly applied for cell-free applications but not for cell biology even though the latter field benefits from nanomaterials as demonstrated with canonical nucleic acids.^7^, ^8^

One class of chemically modified DNA nanostructures of potential in cell biology are membrane-spanning DNA nanopores. In general, engineered nanopores that facilitate transmembrane flux^9^ can be used for cell permeabilization, drug delivery,^10^ but also biosensing.^11^–^13^ In the latter, label-free analytical strategy, individual molecules passing or binding inside a nanoscale pore are detected based on the associated changes in the ionic pore current. A wide range of analytes can be sensed,^11^, ^14^ and DNA can be sequenced by threading individual strands through the pore.^12^, ^15^ Nanopores have traditionally been constructed with re-engineered or de-novo protein scaffolds, or organic synthetic building blocks.^16^ Nanopores composed of folded DNA are the most recent category.^17^–^20^ Formed either by scaffold and staple strands^17^ or short oligonucleotides,^18^, ^19^ the highly negatively charged DNA nanopores insert into hydrophobic bilayer membranes by chemical lipid anchors, whereby cholesterol,^17^ porphyrin,^19^ and a belt of EP tags^18^ were successfully tested. In light of their established ability to span reconstituted membranes, we postulated that the pores could be adapted and exploited to puncture biological cell membranes. Our interest was spiked by the prospect of rationally designing DNA nanostructures for cell biological applications, such as gene transfection, drug permeabilization or targeted killing of diseased cells.

Here we examine whether a membrane-spanning DNA nanopore can interact with cancer cells and potentially trigger cell death (Figure 1 a and b). Our nanopore was composed of a bundle of six DNA duplexes folded from six DNA strands (see Figure S1 in the Supporting Information). The hollow nanobarrel has a channel width of approximately 2 nm, an outer diameter of 5.5 nm, and a height of 14 nm. At the bottom, the pore wall features a 2 nm-high hydrophobic belt composed of 72 EP modifications for membrane insertion. The charge-neutral EP groups replace the native negative phosphate groups of the DNA backbone. In a previous study, the EP belt was successfully used by placing it in the middle of a membrane-spanning DNA nanopore.^18^ In the present report, the belt is moved to the pore terminus to further facilitate membrane insertion.

**Figure 1:**
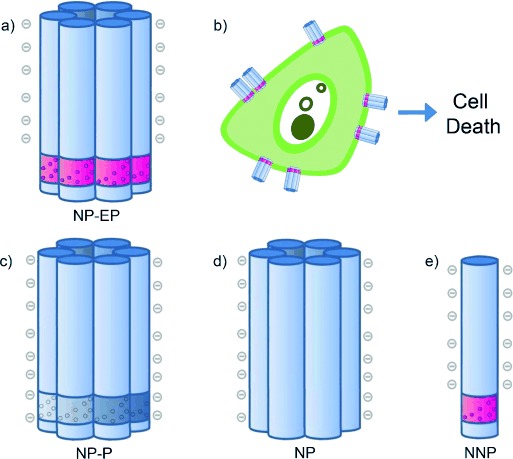
A membrane-spanning DNA nanopore with cytotoxic activity and three nanostructures which serve as negative controls. a) The NP-EP pore is a six-duplex bundle (blue) and contains a hydrophobic belt (purple) made up of 72 ethyl phosphorothioate (EP) groups. b) NP-EP pores insert into a cellular bilayer resulting in cell death. c) NP-P and the two following constructs are not expected to form a pore in the membrane. NP-P features phosphorothioate groups but no ethyl modification. d) NP contains native phosphate groups. e) Construct NNP with EP groups lacks three of the six strands required to generate the six-duplex bundle nanopore. The graphical representation of NPP is simplified, and a complete structure is shown in Figure S4. The nanostructures are not drawn to scale.

In addition to this pore, termed NP-EP, we built three other nanostructures to prove that the EP-belt is essential for cytotoxic activity. These negative controls include barrel NP-P (Figure 1 c, Figure S2) which contains non-modified phosphorothioate groups of negative charge under our experimental conditions of pH 8.0, and barrel NP with an all-native phosphate backbone (Figure 1 d, Figure S3). As a final control, we tested NNP (Figure 1 e, Figure S4) which contains half the set of EP-modified DNA strands and cannot form a complete nanopore.

All constructs were assembled by heating and cooling an equimolar mixture of six DNA oligonucleotides at a concentration of 1 μm in PBS buffer. For the incomplete half-barrel structure NNP, only three strands were used. The sequences of all oligonucleotides are provided in the Supporting Information in Table S1. The DNA strands for NP and NP-P, respectively, were used as supplied by a commercial vendor. By contrast, nucleic acids for barrels NP-EP and NNP were prepared by chemically modifying phosphorothioate with ethyl-iodide to obtain the charge-neutralized EP group following a published procedure.^18^ Accompanying polyacrylamide gel electrophoretic (PAGE) analysis confirmed that treatment with ethyl iodide resulted in completely alkylated phosphorothioate-strands (Figure S5).^18^

Several analytical techniques were applied to confirm the successful formation of nanobarrels NP-EP, NP-P, and NP, and construct NNP. Native agarose gel electrophoresis demonstrated the assembly into a uniform structure. In line with expectations, the NP and NP-P barrels migrated at the same height while the incomplete and smaller NNP structure moved faster (Figure 2 a). Target pore NP-EP with the hydrophobic belt formed a streaky band (Figure 2 a) as found for other hydrophobically tagged nanostructures.^17^, ^19^, ^21^ The streaking likely stems from the interaction with the gel matrix but was largely avoided in sodium dodecyl sulfate PAGE analysis resulting in a sharp band (Figure 2 b). The surprising stability of the DNA structures under these usually denaturing conditions was achieved by running the gel at 8 °C. The migration of assembly products at different heights (Figure 2 b) can be caused by the smaller size (NNP) or the different chemical compositions at the terminal belt (NP). Atomic force microscopy (AFM) analysis established the dimensions of the nanobarrel (Figure 2 c). The apparent height of 2.4±0.5 nm (*n*=20) was expected for tip-compressed hollow DNA nanostructures.^22^ The AFM-derived length and width of 13.0±2.7 nm and 6.9±1.5 nm (full-width-at-half-maximum), respectively, were in good agreement with the theoretical dimensions (14 nm and 5.5 nm). The experimental dimensions reported here are smaller than of similarly sized pores of previous studies^18^, ^19^ because the present AFM read-out examined densely packed barrels (Figure 2 c) which is different to the isolated structures of the preceding reports.

**Figure 2:**
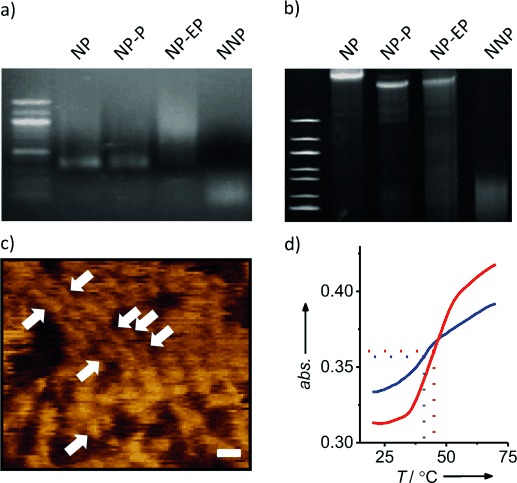
Characterization of pores NP-EP and nanostructures NP-P, NP, and NNP, assembled from DNA oligonucleotides. a) 1.1 % native agarose gel electrophoresis. Left: 100 bp marker (bp=base pair). b) 12 % SDS-PAGE. Left: 100 bp marker. c) AFM analysis of NP. Scale bar 20 nm. d) UV-melting profile of NP-PE with (red) and without (blue) SUVs.

We investigated whether NP-EP interacts with lipid bilayers by monitoring the UV-melting profiles of the DNA. Membrane insertion of the nanopores containing hydrophobic groups should be thermally stabilized upon bilayer anchorage, resulting in an increase in the melting temperature (*T*_m_) of the DNA nanopore.^19^, ^23^ Indeed, for NP-EP with a hydrophobic belt the *T*_m_ increased from 42.5±0.9 °C to 47.3±2.1 °C upon addition of small unilamellar vesicles (SUVs; Figure 2 d). In contrast, *T*_m_ values for barrels NP-P and NP lacking the hydrophobic belt were not influenced by lipid bilayers, implying that these controls do not bind or insert into the SUV bilayers (Table S2).

To study whether DNA nanopores interact with cellular membranes and exert a cytotoxic effect, NP-EP and the three DNA constructs were incubated with cervical cancer cells (HeLa) and assayed for mitochondrial activity which is an indicator of cell viability.^24^ After incubation for 24 h, NP-EP nanopores at a concentration of 60 μg mL^−1^ attenuated cell viability by 20 % compared to the three non-pore forming controls NP, NP-P and NNP (Figure 3 a, 24 h; Figure S6 shows the data for the other concentrations). The cell killing effect of NP-EP was retained up to 72 h after nanopore incubation (Figure S6). A decrease in cell viability of 20 % at a nanopore concentration of approximately 100 nm compares very favorably with synthetic and natural small peptide-based membrane disrupting agents^25^ but is lower than the evolved potency of natural protein pore-forming cytotoxins.^26^–^29^

**Figure 3:**
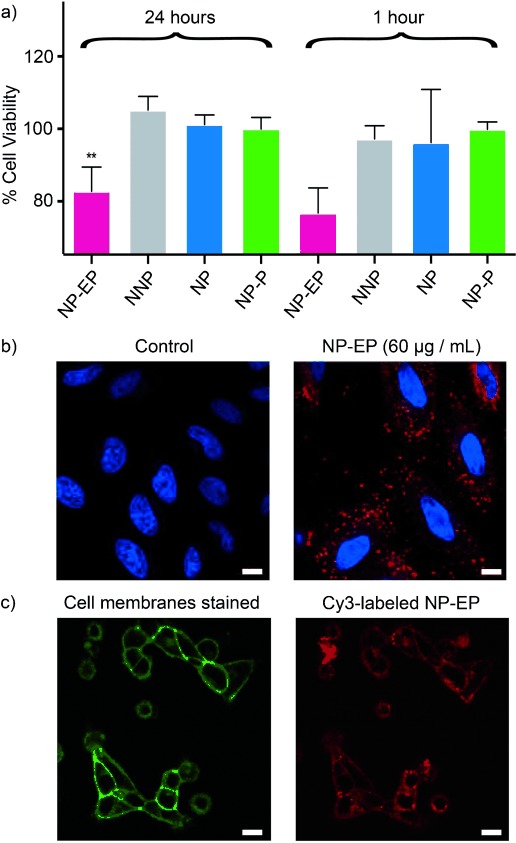
DNA nanopores (NP-EP) are selectively cytotoxic to cervical cancer cells, compared to the three non-membrane-targeting DNA nanostructures. a) AlamarBlue assay depicting cell viability at 24 h and 1 hour after incubation with DNA nanostructure at 60 μg mL^−1^ final concentration. The data represent the averages and means of three independent experiments. ** Statistically significant; *p*<0.01, one-way ANOVA corrected for multiple comparisons using Tukey’s method. b) DNA nanopores interact with cell membranes and enter cancer cells. Confocal microscopy images portraying cell nuclei in blue and DNA nanopores in red. Scale bar: 20 μm. c) NP-EP nanopores co-localize with cell membrane as shown by confocal images portraying cells stained with CellMask Green (left) and the same cells incubated with Cy3-labeled NP-EP pores (right). Scale bar: 50 μm.

To elucidate the mechanism accounting for the observed cytotoxicity, NP-EP pores and the three controls were fluorescently labelled (Table S1) and added to cells. Characterization with flow cytometry showed that NP-EP pores were associated with cells, in line with expectations (Figure S7). The other constructs were also cell-associated. Confocal laser scanning microscopy established that the NP-EP pores were distributed mainly at the cellular membrane (Figure 3 b). By contrast, the three controls were mostly internalized (Figure S8). Internalization has been observed for several other non-membrane DNA nanoconstructs and is likely mediated via the endosome pathway.^8^ The two different types of cellular distribution for our DNA structures indicate that NP-EP pores target membranes while the non-membraneous controls remain water-solubilized. Incubating cells with higher DNA concentrations led to less uptake (Figures S7 and S8), possibly due to a higher tendency of DNA nanostructures to aggregate.

Additional evidence for the specific interaction of the target NP-EP nanopores with cellular membranes was provided by fluorescence microscopy. As expected for membrane pores, Cy3-labeled NP-EP co-localized with cell membranes stained with CellMask Green (Figure 3 c, Figure S9). As further support, insertion of NP-EP pores into in vitro bilayers was established with nanopore recordings (data not shown) in line with the successful electrical measurements of two structurally closely related nanopores.^18^, ^19^

To confirm that membrane association of NP-EP cause cytotoxicity, we examined the short-term kinetics of cell viability, postulating that membrane interaction with the pore-forming nanostructures would lead to rapid cell death. Indeed, viability assays revealed that the cytotoxic effect occurred within an hour of nanopore addition (Figure 3 a; 1 h, NP-EP). Such fast kinetic behavior is similar to that of barrel-forming toxins from bacteria^27^, ^28^ or eukaryotic sources.^26^ These toxins induce membrane damage by creating a transmembrane pore and allowing the influx/efflux of critical ions, nutrients, and second messengers.^28^, ^29^ We are not certain whether the cytotoxic effect of our DNA nanopore is mediated by the same type of mechanism or by a more general membrane perturbation. While these questions about the mechanism will be investigated in follow-up studies, our controls clearly demonstrate that cell death only occurs in the combined presence of a pore and a hydrophobic belt, strongly suggesting that membrane interaction is the reason for cytotoxic activity.

In summary, our DNA nanopores are the first DNA nanostructures to cause the killing of biological cells by targeting cellular membranes. A bilayer-spanning hydrophobic belt composed of ethylated phosphorothioate groups on the outside of pore with 2 nm inner width was key to achieve cell death. The cytotoxic nanopores have the potential to be used as novel and valuable research tools and anti-cancer agents. Their potency could be increased by attaining cancer-type specificity, for example, by tethering to the pore DNA aptamers which recognize cell markers. Additionally, chemical toxins such as DNA-intercalating doxorubicin may be included to enhance the chemotoxic effect. Optionally, more and additional hydrophobic tags may be employed to increase membrane anchorage. Pores might also be engineered to change their cytotoxicity in response to chemical or biochemical stimuli to tune their bioactivity. We expect that DNA nanopores can help create a new class of custom-designed chemical tools for biology and biomedicine.

## Experimental Section

Design of the DNA nanopore and the three constructs: Nanostructures were designed using the caDNAno software.^30^ Several suggested scaffold and staple strands were terminally linked to form a more stable structure composed of only six DNA strands (Figure S1). Using a molecular model generated with Macromodel in combination with caDNAno, the positions for EP modification were selected. For fluorescence imaging, two Cy3 fluorophores were incorporated into the two non-PPT modified strands at the 5′ positions.

Synthesis of EP-modified DNA and nanopore assembly: Phosphorothioate DNA oligonucleotides (5 nanomoles, PAGE-purified, IDT-DNA, Coralville, IO; Table S1) were dissolved in 90 % DMF and 10 % 30 mm Tris-HCl pH 8.0 (25 μL). Iodoethane (5 μL), which had been purified by filtration over a silica gel column, was added to the solution. The mixture was heated to 65 °C for 1.5 h in a screw-top vial, after which the solvent was removed under reduced pressure. The resulting dry solid was dissolved in 0.1 m EDTA pH 8.0 (100 μL) by heating to 90 °C for 5 minutes with vigorous stirring. The DNA was desalted using a NAP-25 column (GE Healthcare). Fractions containing DNA were identified by monitoring absorption at 260 nm and then combined and concentrated under reduced pressure for subsequent nanopore assembly. NP-EP pores were assembled by heating at 95 °C for 5 minutes an equimolar mixture of 4 EP-modified and 2 native strands (Figure S1, Table S1) at 1 μm each dissolved in PBS buffer, followed by cooling to 16 °C at a rate of 0.5 °C min^−1^ in a Varian Cary 300 Bio UV/Vis spectrophotometer equipped with a Peltier cooling element. The other three constructs were assembled in the same way using oligonucleotides with ethyl phosphorothioate groups, phosphorothioate groups or a native backbone as detailed in Table S1 and Figures S2–S4.

Characterization of DNA nanostructures with native and SDS gel electrophoresis, UV spectroscopy, and atomic force microscopy: The assembled DNA structures were analyzed under native conditions using 1.1 % agarose gel electrophoresis in standard TBE buffer supplemented with 11 mm MgCl_2_ and 0.5 μm ethidium bromide, run at 70 V for 60 minutes at 8 °C. For analysis in the presence of SDS, a 12 % polyacrylamide gel with 6 % stack and running buffer 25 mm Tris pH 8.8 supplemented with 0.1 % SDS. DNA (6 pmol) was mixed with 6 μL of 6× gel loading buffer and then loaded into the wells. The electrophoresis conditions were 160 V, 60 minutes, and 8 °C. The bands were visualized by staining with ethidium bromide solution followed by UV illumination. For UV melting point analysis at 260 nm, samples with a concentration of 75 nm dissolved in 0.3 m KCl, 15 mm Tris pH 8.0 were heated at a rate of 1 °C per minute in the spectrophotometer. Melting analysis with small unilamellar vesicles (SUVs) was carried out at a molar ratio of 0.1 to 500 for nanopores to SUV lipid. The vesicles were prepared by sonication of a lipid solution containing DPhPC and cholesterol. DPhPC (11.1 mM, 2.65 mL) was added to cholesterol (10 mM, 0.295 mL) in a 20 mL round bottom flask. The solution was dried under vacuum using a rotary evaporator for 30 minutes, followed by an additional 4 hours under ultrahigh vacuum. Deionized water (500 µL) was added to the thin film, and the suspension was sonicated for 10–20 minutes. The SUVs with a size range of 50–100 nm, as determined by dynamic light scattering, were stored at 4 °C for up to a week. AFM analysis was carried out by first adsorbing NP DNA barrels onto mica following a modified version of a published procedure.^31^ Freshly cleaved mica was incubated with a solution of 150 mm KCl and 7.5 mm Tris pH 8.0. The DNA-barrel solution was added to give a final DNA concentration of 20 nm. AFM topographical images were acquired in situ at RT with a multimode atomic force microscope as described.^18^

Cell culture: Human cervical cancer cells (HeLa cells) were a kind gift from Prof. Charles Swanton (UCL, UK). Cells were grown in Dulbecco’s modified Eagle Medium (DMEM) supplemented with 10 % heat inactivated fetal bovine serum, 50 μg mL^−1^ streptomycin, and 50 IU mL^−1^ penicillin. Cells were passaged regularly when reaching 80 % confluence.

Cell viability: HeLa cells were seeded at a density of 1500 cells per well, in a 96 well plate. After 24 h, DNA nanostructures were incubated with cells at the desired concentrations in triplicate. At the chosen time-points, the AlamarBlue reagent was added to cells for 2 1/2 h, as recommended by the manufacturer’s guidelines.^24^ Absorbance readings were recorded and normalized to those of untreated control cells.

Live cell imaging: HeLa cells were plated at a density of 15 000 cells per well, in an ibidi chamber slide (Vitaris, France). The following day, Cy3-labeled DNA nanostructures were added to cells at the desired concentrations. After incubation at 37 °C for 3 h, cells were washed three times with DMEM to remove non-internalized DNA. For staining of cellular nuclei, a solution of Hoechst 33258 pentahydrate was added to a final concentration of 3 μg mL^−1^ followed by incubation for 20 minutes at 37 °C. Cells were washed and supplanted with fresh media prior to imaging on a LSM 700 inverted confocal microscope (Carl Zeiss, Germany). Imaging was achieved using a 63× objective lens and laser excitations of 405 nm (Hoechst channel) and 555 nm (Cy3 channel).

Flow cytometry: HeLa cells were plated in duplicate at a density of 45 000 cells per well in a 24 well plate. After 24 h, cells were incubated with DNA structures at the desired concentration at 37 °C for 3 h. Cells were washed three times with media and once with PBS, and harvested by centrifugation at 300 g for 5 minutes. Supernatants were discarded and cells were resuspended in fresh media and filtered into FACS tubes (BD Biosciences, USA). 10 000 live events were recorded for each sample using a LSRFortessa machine (BD Biosciences) equipped with a 488 nm blue laser. The data was analyzed with FlowJo software (TreeStar Inc., USA).

Membrane colocalization**:** HeLa cells were plated at a density of 15 000 cells per well, in an ibidi chamber slide. The following day, Cy3-labeled DNA nanostructures were added to cells at the desired concentrations and incubated at 37 °C for 20 minutes. The cells were washed three times with media to remove adherent non-internalized DNA. To stain cell membranes, a solution of CellMask Green (1×) was added to cells and incubated 37 °C for 15 minutes followed by washing with fresh media prior to remove excess dye. Imaging was achieved using laser excitation at 488 nm (CellMask Green channel) and 555 nm (Cy3 channel). Co-locolization analysis was accomplished using the Coloc 2 plug-in of Fiji, a platform of ImageJ (NIH, USA).^32^
