# Supplementary material for: Membrane-Spanning DNA Nanopores with Cytotoxic Effect
Source: Angew Chem Int Ed Engl. 2014 Oct 7;53(46):12466–70. doi: 10.1002/anie.201405719 (PMC4282124; doi:10.1002/anie.201405719)
Supplement: Supplementary file 1 [file anie0053-12466-sd1.pdf]

Angewandte Chemie
Eine Zeitschrift der Gesellschaft Deutscher Chemiker

Supporting Information

© Wiley-VCH 2014

69451 Weinheim, Germany

Membrane-Spanning DNA Nanopores with Cytotoxic Effect**

*Jonathan R. Burns, Noura Al-Juffali, Sam M. Janes, and Stefan Howorka**

anie_201405719_sm_miscellaneous_information.pdf

Contents

1. Experimental Details 3
1.1. DNA sequences 3
1.2. 2D maps of DNA nanobarrels 4
2. Experimental Results 6
2.1. Alkylation of phosphorothioate DNA 6
2.2. UV melting point analysis of DNA nanostructures 6
2.3. Cell biological analysis 7

1. Experimental Details

1.1. DNA sequences

Table S1. Sequences of DNA oligonucleotides used to generate DNA nanopore NP-EP, nanobarrels NP-P and NP, and construct NNP.

#	Sequences (5' → 3')
1*	ACA*G*G*A*T*T*TTCGCCTGCTGGGGCAAACCAGCGTGGACCGCTTTTTTGGCTATTCTTTTGATTTATAAGGGATTTTGCCGA*T*T*T*C*G*GAA
1	ACAGGATTTTCGCCTGCTGGGGCAAACCAGCGTGGACCGCTTTTTTGGCTATTCTTTTGATTTATAAGGGATTTTGCCGATTTCGGAA
2*	CAACTCTCTCAGGGCCAGGCGGTGAAGGGCAATC*A*G*C*T*G*TTGTTTTCAA*C*A*G*C*A*T*C*C*TGTTTC*C*G*A*A*A*TCGGCATTAAAG*A*C*CAGCTG
2	CAACTCTCTCAGGGCCAGGCGGTGAAGGGCAATCAGCTGTTGTTTTCAACAGCATCCTGTTTCCGAAATCGGCATTAAAGACCAGCTG
3*	TCT*C*A*C*T*G*GTGAAAAGAAAAACCACCCTGGCGCCCAATACGCTTTTTCCCCGCGCGTTGGCCGATTCATTAATGCAGCTG*G*C*A*C*G*ACA
3	TCTCACTGGTGAAAAGAAAAACCACCCTGGCGCCCAATACGCTTTTTCCCCGCGCGTTGGCCGATTCATTAATGCAGCTGGCACGACA
4*	GGCGAA*A*T*GATTGCTTTCAC*C*A*G*T*G*AGATGT*C*G*T*G*A*C*G*T*GGATTTTTCC*A*C*G*T*T*CTTTAATAGTGGACTCTTGTTCCAAACTGGAACA
4	GGCGAAATGATTGCTTTCACCAGTGAGATGTCGTGACGTGGATTTTTCCACGTTCTTTAATAGTGGACTCTTGTTCCAAACTGGAACA
5	TGTTCCAAATAGCCAAGCGGTCCACGCTCCCTGAGGGGCGCCAGGGTGGGAATCGGACAAGAGTCCACTAAAATCCCCCCAGCA
6	CATTAATTTTTTCTCCTTCACCGCCTGGGGTTTGCTTATAAATCAAAAGGTTTGGACCAACGCGCGGGGAGCGTATTAGAGTTG

* = phosphorothioate (PPT) modification. Note: Instead of strand 2* with a length of 88 nt, a combination of a 21-mer and a 67-mer strand was optionally used to obtain a higher quantity from the commercial vendor of the DNA strands. The 21-mer and 67-mer correspond to the 5'- and 3'-terminal sequence, respectively, of strand 2*. NP-EP pores generated either with 2* or the 21-mer and 67-mer showed no difference in terms of electrophoretic mobility in native agarose and SDS-PAGE gels, UV-melting temperature, and cell biological effect.

1.2. 2D maps of DNA nanobarrels

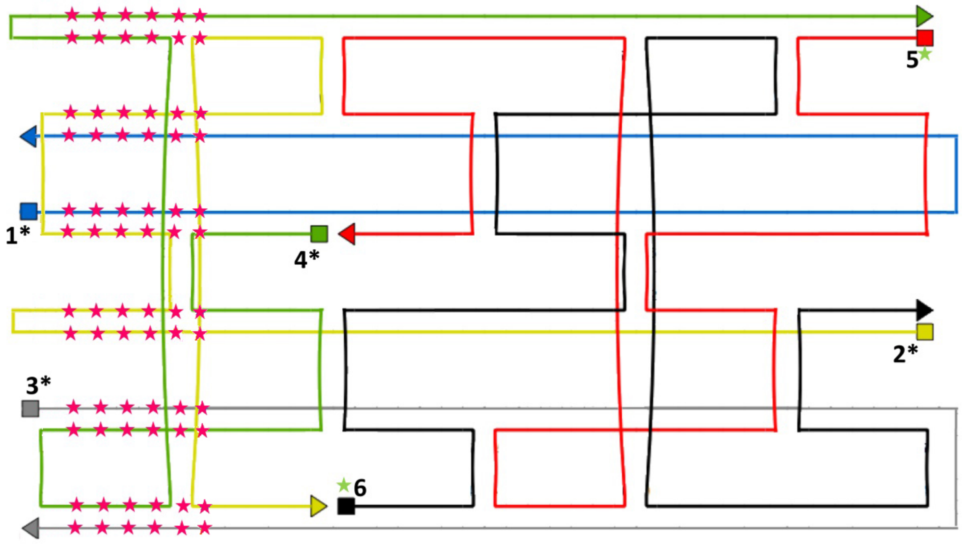

Figure S1. 2D map of NP-EP composed of DNA strands 1*, 2*, 3*, 4*, 5 and 6, as indicated by numbers. Strands 1*, 2*, 3*, and 4* carry ethyl modified PPT-groups as indicated by purple stars. The 5' and 3' termini of the DNA strands are represented by a square and triangle, respectively. Strands 2* and 4* contain a hairpin of TTTT to prevent blunt-end stacking. Similarly, 1* and 3* contain four additional T at the cross-over. The green stars represent the position of a Cy3 fluorophore labeled uridine base. The position is the same for the following three DNA nanoconstructs.

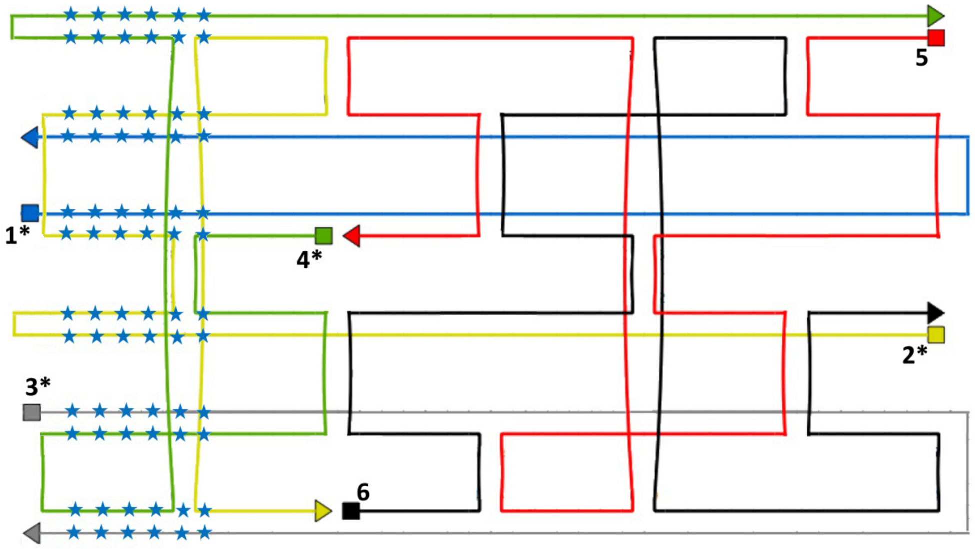

Figure S2. 2D map of NP-P composed of PPT-modified strands 1*, 2*, 3*, and 4*, and DNA strands 5 and 6 with a native phosphodiester backbone. Each blue star represents a PPT modification.

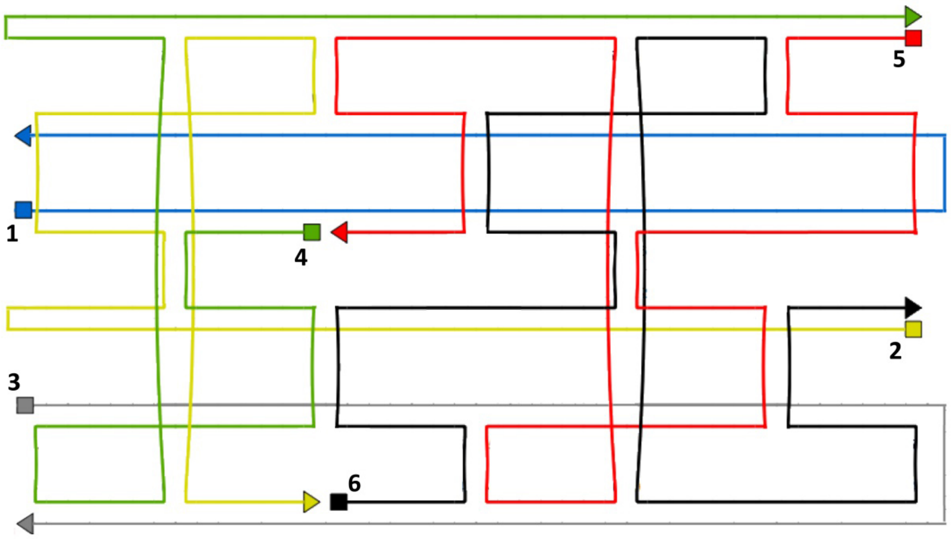

Figure S3. 2D map of NP composed of strands 1-6 carrying a native phosphodiester backbone.

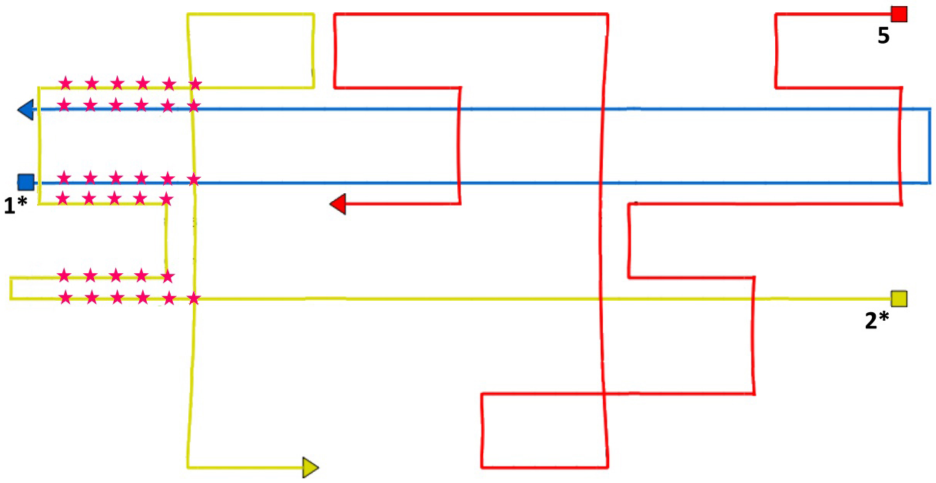

Figure S4. 2D map of NNP composed of native DNA strand 5 and strands 1* and 2* which carry ethyl modified PPT groups as indicated with purple stars.

2. Experimental Results

2.1. Alkylation of phosphorothioate DNA

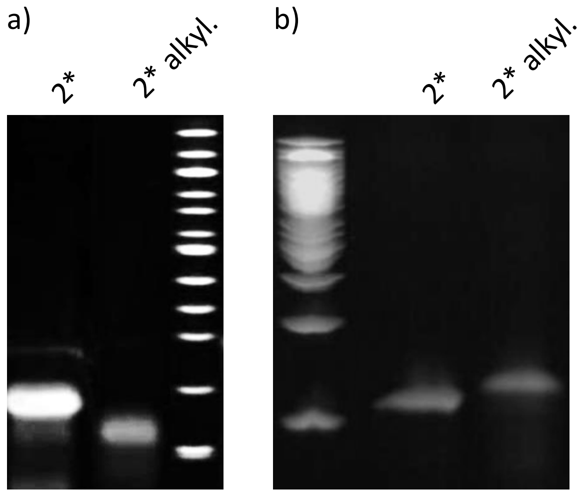

Figure S5. Monitoring the alkylation of PPT-modified DNA strand 2*. a) 12 % SDS-PAGE analysis run for 60 min at 160 volts at 8 °C with 0.1 nmole of DNA loaded per lane. 50 bp marker. b) 6 M urea PAGE of PPT and PPT-Et modified DNA run for 45 mins at 200 volts at room temperature with 0.1 nmole of DNA loaded per lane. 100 bp marker.

2.2. UV melting point analysis of DNA nanostructures

Table S2. T_m values of DNA nanopore NP-EP and nanobarrels NP-P and NP with and without SUVs.

	Melting temperature, T_m / °C	
Nanopore	without SUVs	with SUVs
NP-EP	42.5	47.3
NP-P	50.0	49.3
NP	51.2	52.4

2.3. Cell biological analysis

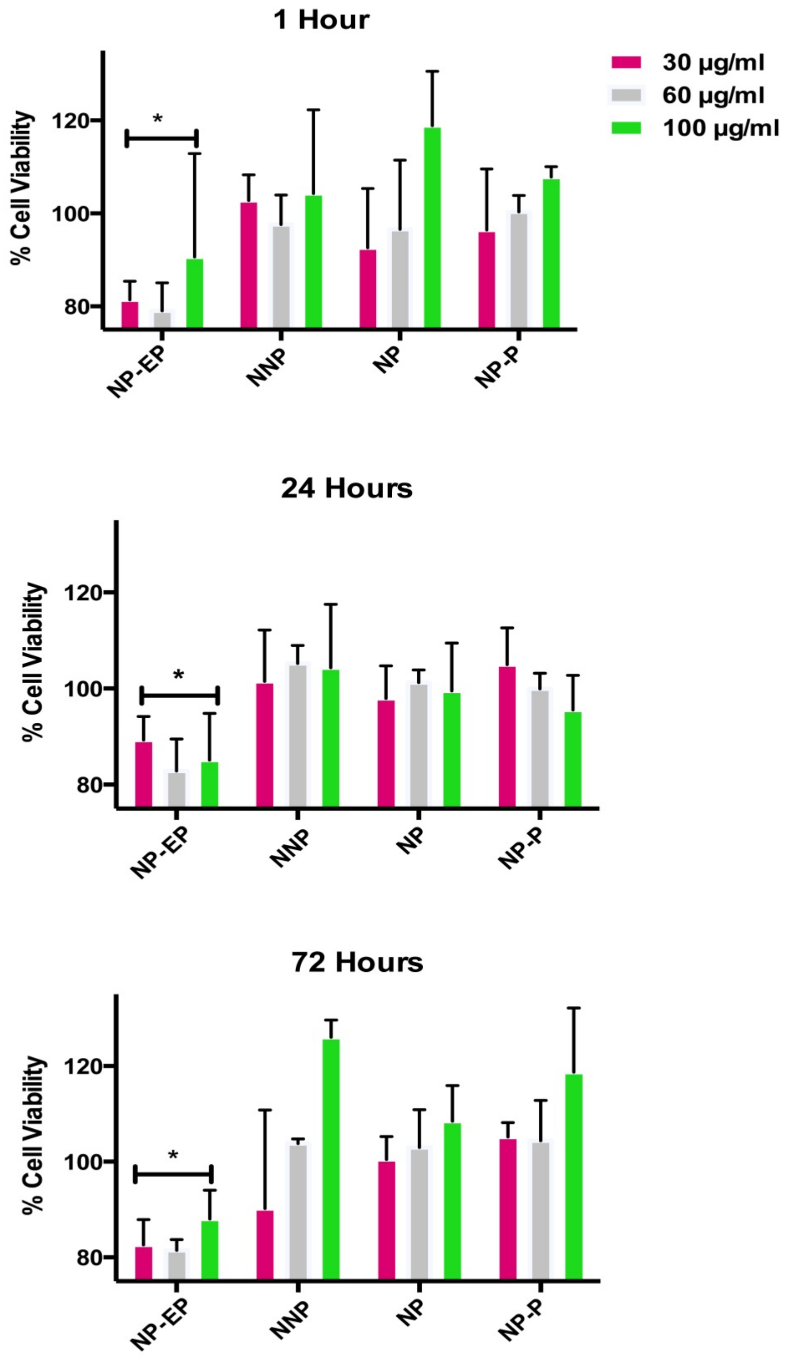

Figure S6. NP-EP DNA nanopores carrying a hydrophobic belt are selectively cytotoxic to cervical cancer cells, compared to other DNA nanostructures NP-P, NP, and NNP which do not contain a hydrophobic belt. Alamar Blue assay depicting cell viability at 1 hour, 24 hours, and 72 hours after incubation with the DNA nanostructures. * Statistically significant, $p<0.05$, 2-way ANOVA followed by Tukey's correction for multiple comparisons. All values are normalized to untreated control cells. The concentrations used for NP-EP, NP, and NP-P were as stated. For NNP, the used concentration was half the given value.

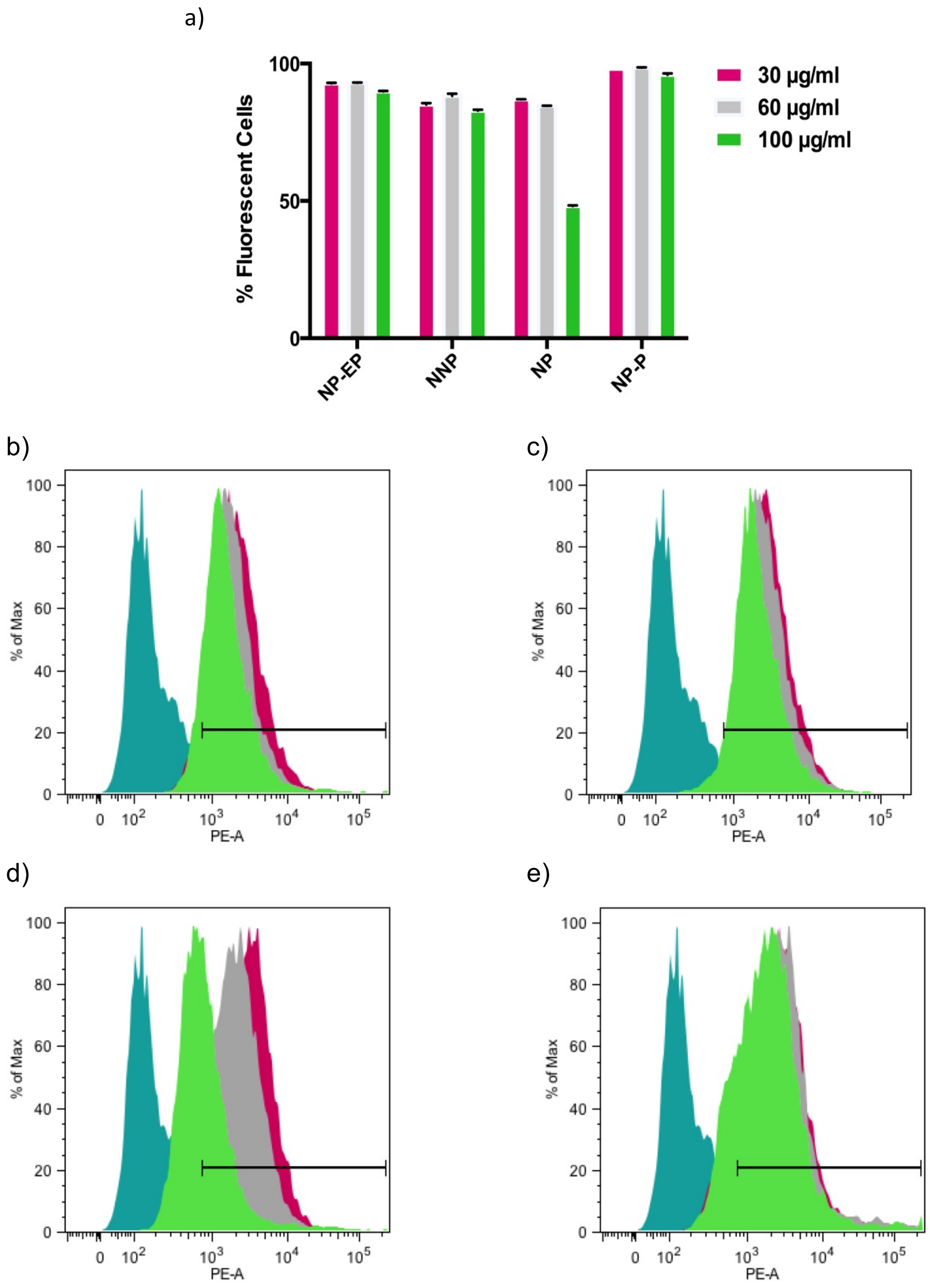

Figure S7. Flow cytometry data summarizing the cellular association of Cy3-labeled DNA nanostructures. a) Percentage of fluorescent cells. b-e) Semilogarithmic plots of cell count vs. fluorescence intensity as recorded in the PE-A channel (Cy3). b) NP-EP, c) NP-P, d) NP, e) NNP. The plots in b)-e) are color-coded as in a). Turquoise indicates the blank sample without addition of DNA. The concentrations used for NP-EP, NP, and NP-P were as stated. For NNP, the used concentration was half the given value. The data for 100 µg/ml of NP in a) is markedly lower than the other data points, mostly likely because DNA nanopores can aggregate at high concentrations, are not taken up by cells, and are then removed by washing after the incubation step.

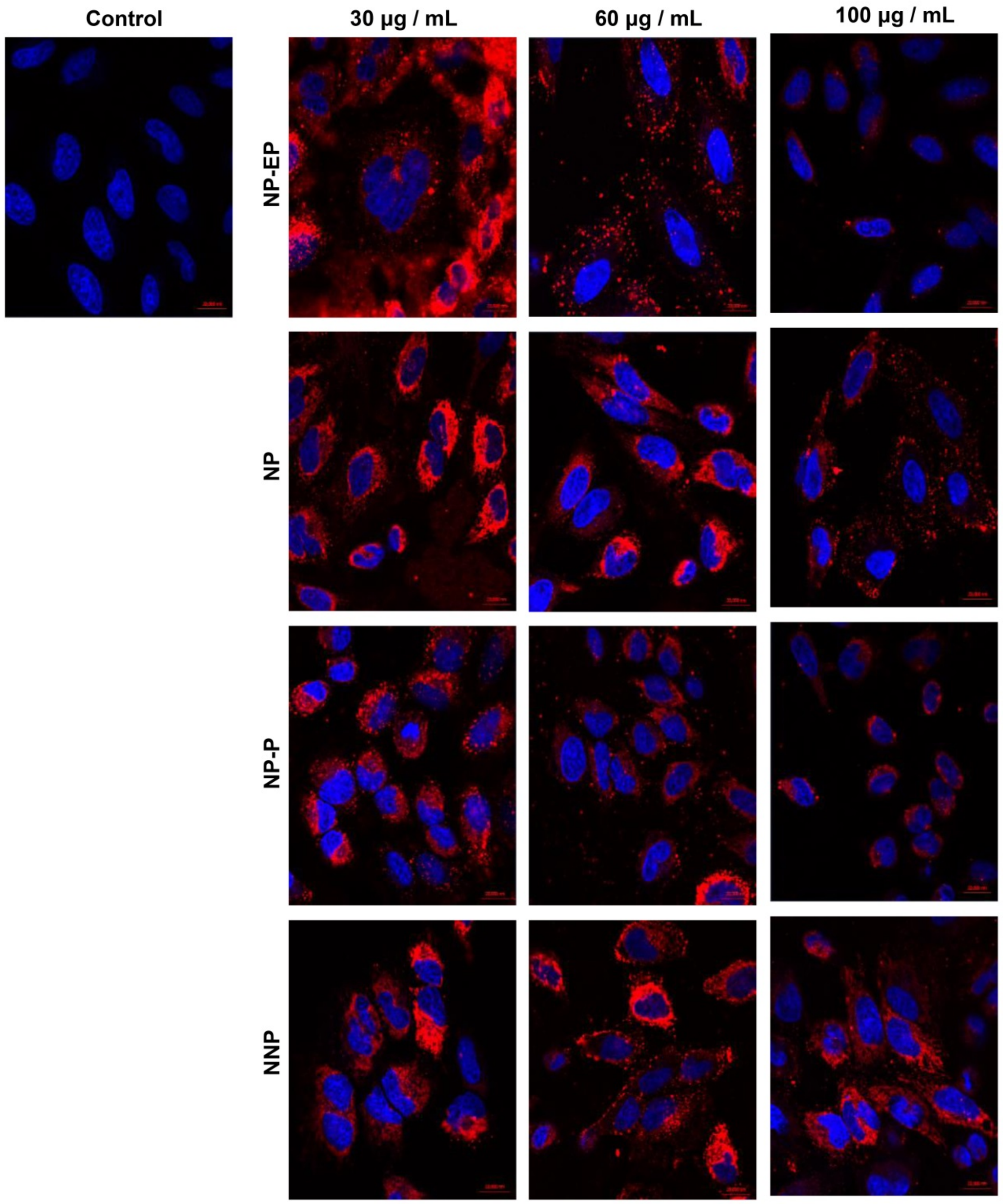

Figure S8. DNA nanopore NP-EP, nanobarrels NP-P and NP, and construct NNP interact with cell membranes and enter HeLa cancer cells. Confocal microscopy images portraying cell nuclei in blue (Hoechst 33258) and DNA structures in red (Cy3). DNA nanopore NP-EP localizes more peripherally on cell surfaces at 60-100 µg/ml compared to the perinuclear pattern seen with other DNA nanostructures. Scale bar at bottom right in red, 20 µm. The concentrations used for NP-EP, NP, and NP-P were as stated. For NNP, the used concentration was half the given value.

a)

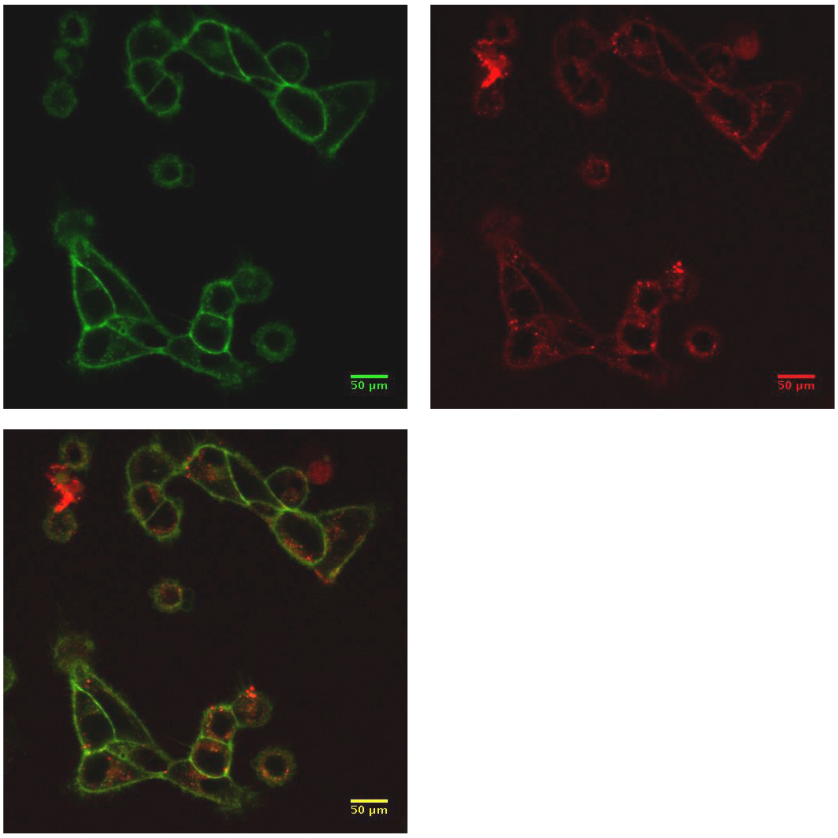

b)

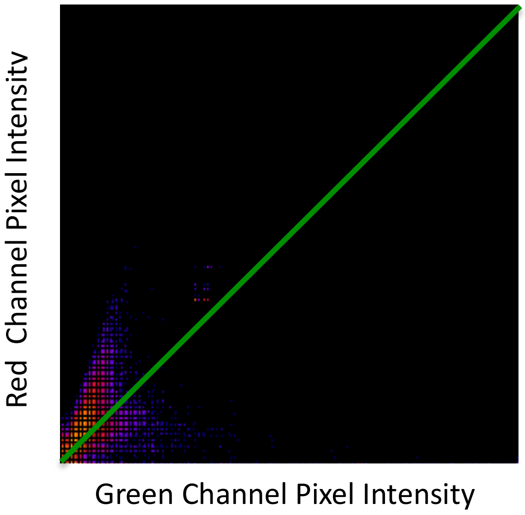

Figure S9. NP-EP nanopores colocalize with the cell membrane. a) Confocal images displaying cells with CellMask™ Green-stained membranes (Alexafluor 488 green channel), and the same cells incubated with Cy3-labeled NP-EP pores (Cy3 red channel), and the merged-channel imaging demonstrating co-localization. Scale bar: 50 µm. b) 2-D intensity histogram depicting co-localization of the green and red channels with perfect co-localization indicated by the green diagonal line.
